# Local delivery of tetramethylpyrazine eliminates the senescent phenotype of bone marrow mesenchymal stromal cells and creates an anti‐inflammatory and angiogenic environment in aging mice

**DOI:** 10.1111/acel.12741

**Published:** 2018-02-28

**Authors:** Bo Gao, Xisheng Lin, Huan Jing, Jing Fan, Chenchen Ji, Qiang Jie, Chao Zheng, Di Wang, Xiaolong Xu, Yaqian Hu, Weiguang Lu, Zhuojing Luo, Liu Yang

**Affiliations:** ^1^ Institute of Orthopedic Surgery Xijing Hospital Fourth Military Medical University Xi'an China; ^2^ State Key Laboratory of Military Stomatology & National Clinical Research Center for Oral Diseases & Shaanxi International Joint Research Center for Oral Diseases Center for Tissue Engineering, School of Stomatology Fourth Military Medical University Xi'an China; ^3^ Department of Neurosurgery Xijing Hospital Fourth Military Medical University Xi'an China; ^4^ Department of Orthopedic Surgery Hong‐Hui Hospital Xi'an Jiaotong University College of Medicine Xi'an China

**Keywords:** bone marrow niche, bone marrow stem/progenitor cells, cellular senescence, EZH2‐H3K27me3, H‐type vessel, tetramethylpyrazine

## Abstract

Aging drives the accumulation of senescent cells (SnCs) including stem/progenitor cells in bone marrow, which contributes to aging‐related bone degenerative pathologies. Local elimination of SnCs has been shown as potential treatment for degenerative diseases. As LepR^+^ mesenchymal stem/progenitor cells (MSPCs) in bone marrow are the major population for forming bone/cartilage and maintaining HSCs niche, whether local elimination of senescent LepR^+^ MSPCs delays aging‐related pathologies and improves local microenvironment need to be well defined. In this study, we performed local delivery of tetramethylpyrazine (TMP) in bone marrow of aging mice, which previously showed to be used for the prevention and treatment of glucocorticoid‐induced osteoporosis (GIOP). We found the increased accumulation of senescent LepR^+^ MSPCs in bone marrow of aging mice, and TMP significantly inhibited the cell senescent phenotype via modulating Ezh2‐H3k27me3. Most importantly, local delivery of TMP improved bone marrow microenvironment and maintained bone homeostasis in aging mice by increasing metabolic and anti‐inflammatory responses, inducing H‐type vessel formation, and maintaining HSCs niche. These findings provide evidence on the mechanisms, characteristics and functions of local elimination of SnCs in bone marrow, as well as the use of TMP as a potential treatment to ameliorate human age‐related skeletal diseases and to promote healthy lifespan.

**Abbreviations**
GIOP: glucocorticoid‐induced osteoporosis
MSPCs: bone marrow stem/progenitor cells
SASP: senescence‐associated secretory phenotype
SA‐βGal: senescence‐associated β‐galactosidase
SnCs: senescent cells
TMP: tetramethylpyrazine

## 1 INTRODUCTION

Aging is the main causative pathological factor for bone degenerative diseases and functional deficits in humans (Finch, 2010; Stenderup, Justesen, Clausen, & Kassem, 2003). During aging, bone homeostasis is interrupted with the chaos of the marrow microenvironment, including a disrupted HSC niche (Mendelson & Frenette, 2014), decreased vessel formation (Kusumbe, Ramasamy, & Adams, 2014) and abnormal inflammation factor release (Lepperdinger, 2011). As a result, increased cellular senescence in bone marrow can be induced by cellular damage or environment changes. It is reported that senescent cells (SnCs) accumulate in bone marrow with aging (Farr et al., 2017) and contribute to age‐related pathologies through their secretion of factors contributing to the senescence‐associated secretory phenotype (SASP) (Campisi, 2000, 2013; Nelson et al., 2012). SnCs exhibit essentially stable cell cycle arrest through the actions of tumour suppressors such as p16^INK4a^, p53, p21^CIP1^ (Campisi, 2005; Serrano, Lin, McCurrach, Beach, & Lowe, 1997) and also include increased lysosomal β‐galactosidase activity, robust secretion of inflammatory cytokines/chemokines, and nuclear foci containing DNA damage response proteins or distinctive heterochromatin. Although cell senescence has been well studied in recent decades, the mechanisms and local treatment targets for SnCs‐induced bone degenerative disease are not well understood.

Mesenchymal stromal cells (MSCs), including mesenchymal stem/progenitor cells (MSPCs), play an essential role in bone metabolism and HSC maintenance (Kfoury & Scadden, 2015). However, the proliferation and function of MSCs are largely impaired during aging (Alt et al., 2012; Fehrer, Laschober, & Lepperdinger, 2006). Bonyadi et al. (2003) reported that MSC “deficiencies” in terms of either number or cellular function could be the driving force of musculoskeletal diseases, and other studies also mentioned the diseases remote from the musculoskeletal environment, including osteoarthritis and diabetes due to MSC “deficiencies” (Jeon et al., 2017; Zang et al., 2017). Likewise, MSC senescence during aging markedly impairs the HSC niche (Iyer, Brooks, Gumbleton, & Kerr, 2015), decreases osteoblast numbers and disrupts epithelial–mesenchymal transition (Zhou et al., 2008). LepR^+^ cells in bone marrow were a major source of CFU‐F‐forming MSPCs in adult and formed bone, cartilage and adipocytes in culture and upon transplantation (Zhou, Yue, Murphy, Peyer, & Morrison, 2014). Additionally, LepR^+^ cells are essential for maintaining the HSC niche through secreting Scf (Asada et al., 2017). However, little is known about whether LepR^+^ cells are senescent and dysfunctional during aging. Increasing in vivo evidence suggests that cellular senescence is modulated by several post‐translational modifications of histones, including methylation, acetylation, phosphorylation and ubiquitination. For instance, Ezh2 is the functional enzymatic component of polycomb repressive complex 2 (PRC2), which has histone methyltransferase activity and primarily trimethylates histone H3 on lysine 27 (Margueron & Reinberg, 2011). Ezh2 has been shown to be recruited to and methylate the Ink4A locus in human BMSCs and to be involved in inhibiting senescence by the Twist‐1 transcription factor (Cakouros et al., 2012). Li et al. (2017) also showed that MSC senescence could be epigenetically modulated by EZH2‐H3K27me3 in late puberty. However, whether aging‐induced MSC senescence and microenvironment disruption could be pharmacologically and epigenetically modulated still need to be determined.

Tetramethylpyrazine (TMP), the bioactive component extracted from Ligusticum wallichii Franchat (Chuanxiong) which is widely used for the treatment of ischaemic stroke, cerebral infarction and degenerative diseases of the central nervous system (Chen et al., 2017; Sun et al., 2012), has been reported to have anti‐inflammatory and anticancer effects in certain cell types (Chen et al., 2014; Gong, Ivanov, Davidson, & Hei, 2015; Yu et al., 2015). We previously showed that TMP could protect MSCs from glucocorticoid‐induced apoptosis and be used for the prevention and treatment of glucocorticoid‐induced osteoporosis (GIOP) (Wang et al., 2017). In this study, we aimed to investigate the local effect of TMP on the bone marrow of aging mice and to determine whether the senescent phenotype of MSCs could be eliminated. Our findings revealed that local delivery of TMP eliminates the senescent phenotype of LepR^+^ MSCs via epigenetically modulating EZH2‐H3K27me3. Moreover, TMP maintains the HSCs niche and creates an anti‐inflammatory and angiogenic environment in aging mice. These findings indicate that TMP could serve in stem cell transplantation and a potential treatment for aging‐induced bone degenerative diseases.

## 2 RESULTS

### 2.1 Clearance of senescent cells by local delivery of tetramethylpyrazine attenuates bone loss and improves the metabolic microenvironment in aging mice

**Figure 1:**
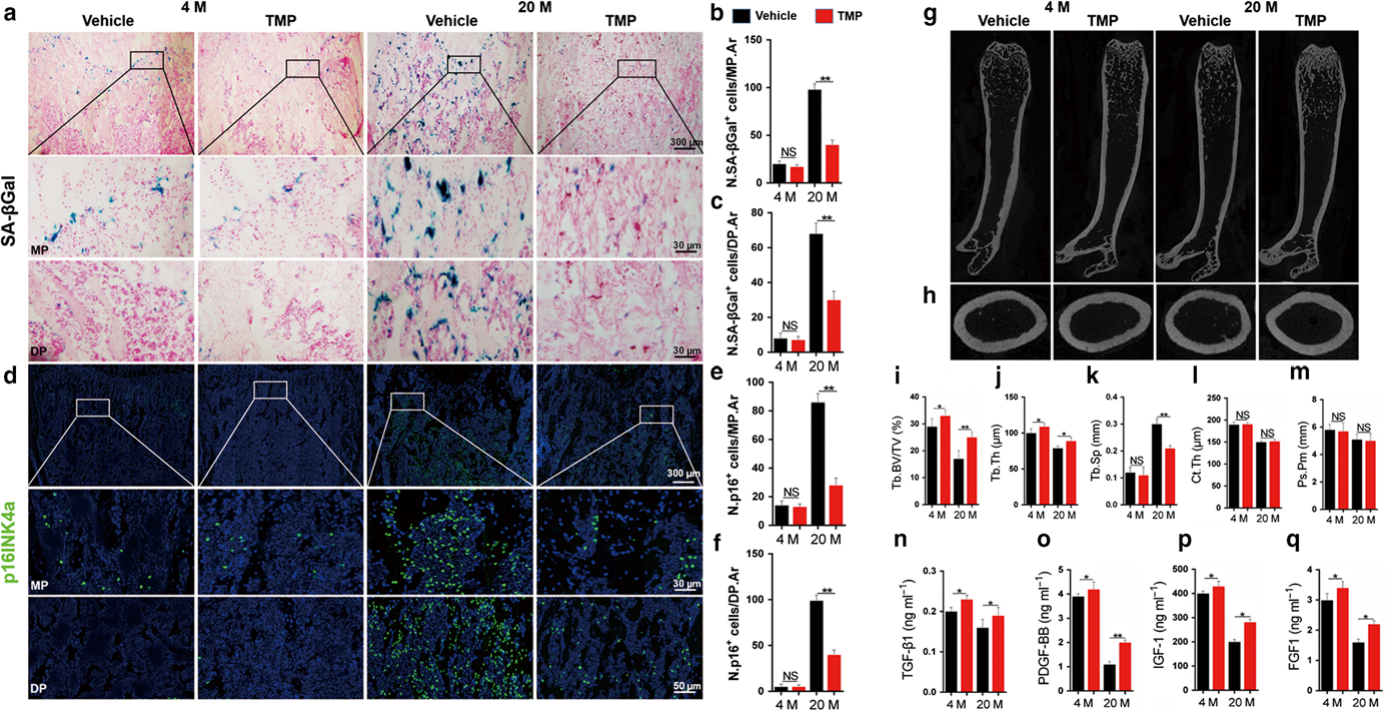
Clearance of senescent cells by local delivery of tetramethylpyrazine attenuates bone loss and improves metabolic microenvironment in aging mice. 4‐ and 20‐month‐old male mice were treated with TMP or vehicle for 8 weeks. (a–c) Representative SA‐βGal staining and quantitative analysis of SA‐βGal^+^ cells in femoral metaphysis (MP) and diaphysis (DP) sections. Images in (a) are lower power with boxes outlining the area of higher power in (MP) and (DP). Numbers of SA‐βGal^+^ cells per mm^2^ tissue area in metaphysis (N. SA‐βGal^+^ cells/MP.Ar) (b) and diaphysis (N. SA‐βGal^+^ cells/DP.Ar) (c). (d–f) Representative images of immunofluorescence staining (d) and quantitative analysis of p16^INK^ ^4a+^ (g) cells in femoral metaphysis (MP) and diaphysis (DP) sections. DAPI stains nuclei blue. Images in (d) are lower power with boxes outlining the area of higher power in (MP) and (DP). Numbers of p16^INK^ ^4a+^ cells per mm^2^ tissue area in metaphysis (N. p16^+^ cells/MP.Ar) (b) and diaphysis (N. p16^+^ cells/DP.Ar). (g–h) Representative microcomputed tomography (μCT) images of femora (g, longitudinal section; h, cross section). (i–m) Quantitative μCT analysis of the trabecular bone fraction (Tb. BV/TV) (i), trabecular bone thickness (Tb. TH) (j), trabecular bone space (Tb. SP) (k), cortical thickness (Ct. Th) (l) and periosteal perimeter (Ps. Pm) (m) of femora. (o–r) ELISA analysis for bone marrow (BM) TGF‐β1 (o), PDGF‐BB (p), IGF‐1 (q) and FGF‐1 (r). *n* = 5. Data are represented as mean ± SEM. MP, metaphysis. DP, diaphysis. *p < .05,**p < .01, NS, no significance as determined by two‐tailed Student's *t* tests

During aging, senescent cells accumulate in various tissues and organs (Campisi, 2005) and disrupt tissue structure and function (Coppe et al., 2008; Rodier & Campisi, 2011). Cells exhibiting a senescent phenotype display essentially stable cell cycle arrest through the actions of tumour suppressors, such as p16^INK4a^, p53, p21^CIP1^ or others (Campisi, 2005; Serrano et al., 1997). We first investigate the local effect of TMP on bone marrow cells of aging mice. We screened the dosage of TMP injected in bone marrow cavity of either 4‐ or 20‐month‐old male mice, and 10 μg/kg was chosen for the following in vivo experiment because of the high efficiency of its antisenescence effect (Figure S1). Additionally, we have showed in previous studies that TMP has no toxic effect on live cells (Wang et al., 2017). We conducted exploratory senescence‐associated β‐galactosidase (SA‐βGal) staining (Figure 1a–c) and p16^INK4a^ staining (Figure 1d–f) in femoral bones of mice at 4 months and 20 months. A significant increase in the number of SA‐βGal^+^ cells and p16^+^ cells was observed in either metaphysis (i.e. trabecular bone adjacent to the growth plate) or diaphysis of the femur in 20‐month‐old male mice relative to 4‐month‐old mice, whereas the SA‐βGal^+^ senescent cells and p16^+^ cells were largely diminished at the same region of femoral bone in TMP‐treated 20‐month‐old male mice (Figure 1a–f). However, SA‐βGal^+^ cells and p16^+^ cells remain unchanged in TMP‐treated 4‐month‐old mice relative to vehicle‐treated controls, indicating that the antisenescence effect of TMP mainly targeted aging conditions (Figure 1b,c,e,f). To investigate whether the p16^+^ cells that increased in number during aging were the same cells observed with increased DNA damage and decreased proliferation. Co‐immunostaining of p16^INK4a^ with gamma‐H2AX showed that the number of p16^+^‐expressing gamma‐H2AX^+^ cells was significantly increased in aging mice, and local delivery of TMP significantly ameliorated the senescent phenotype of the cells (Figure S2A,B). We further quantified the percentage of p16^+^‐expressing gamma‐H2AX^+^ cells to p16 cells; the result showed that nearly 82% of p16^+^ cells suffered from a high DNA damage burden (Figure S2C). Moreover, co‐immunostaining of p16 with BrdU showed that the number of proliferative cells significantly decreased in aging mice and that TMP further ameliorated this phenotype (Figure S2D–F), which indicates that the majority of senescent cells suffered from a high DNA damage burden and markedly lost their proliferation ability and function, and TMP rescued the phenotype via inhibiting cell senescence and promoting proliferation. As a result, TMP significantly increased the trabecular bone microarchitecture (Figure 1g,i,j,k), while the cortical bone was not markedly affected (Figure 1h,l,m). Moreover, TMP significantly decreased osteoclast numbers in trabecular bone (Figure S3A) and the endosteal bone surface (Figure S3B) but not the periosteal bone surface (Figure S3C) and also decreased the circulating CTX levels in 20‐month‐old mice (Figure S3D). Interestingly, we isolated osteoclasts from 4‐ and 20‐month‐old mice and cultured them in osteoclastogenic medium with 50 μm TMP or vehicle. Trap staining and RT‐PCR of osteoclast‐related genes showed that TMP had no direct effect on osteoclast formation and differentiation (Figure S3E–I), which indicates that TMP inhibits osteoclasts in vivo through indirect effects. Of note, local delivery of TMP markedly improved bone marrow TGF‐β1, PDGF‐BB, IGF‐1 and FGF‐1 levels in aging mice, in which the levels of these growth factors were dramatically reduced relative to their levels in 4‐month‐old control mice (Figure 1n–q). Interestingly, TMP also markedly increased the bone marrow levels of these growth factors in 4‐month‐old mice (Figure 1n–q). These findings indicated the essential role of TMP in inhibiting the senescent cell phenotype and improving the bone marrow microenvironment.

### 2.2 Tetramethylpyrazine inhibits the senescent phenotype of LepR^+^ bone marrow stem/progenitor cells in aging mice

**Figure 2:**
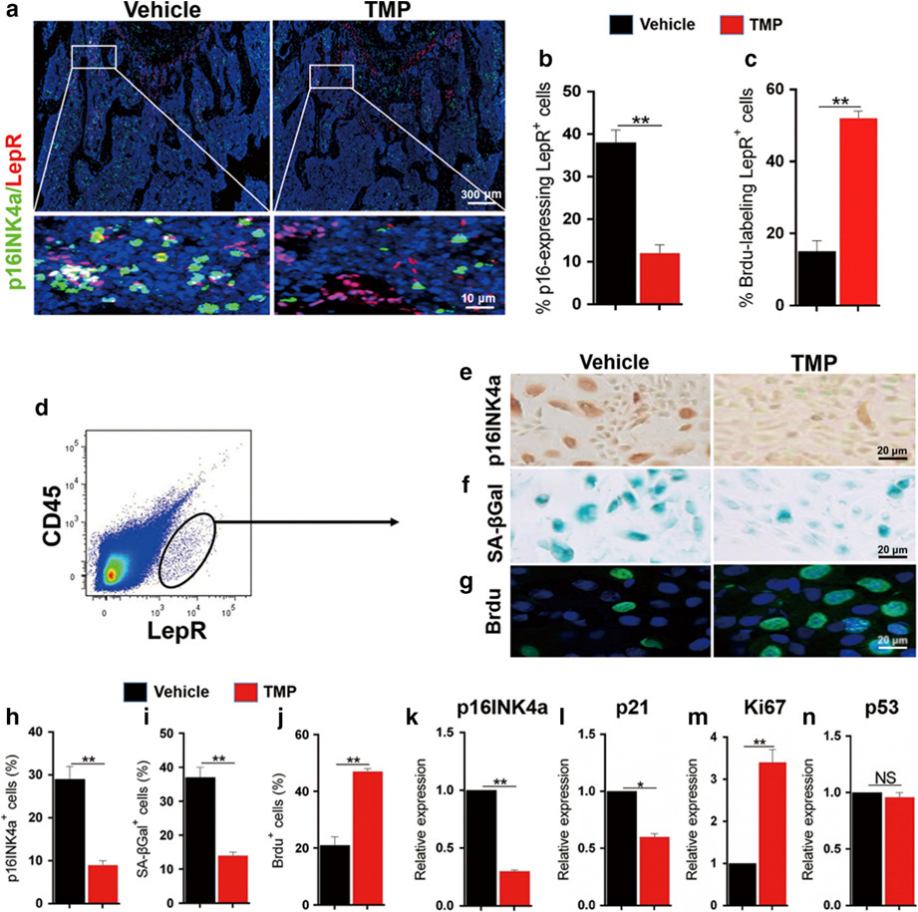
Tetramethylpyrazine inhibits the senescent phenotype of LepR^+^ bone marrow stem/progenitor cells in aging mice. Twenty‐month‐old male mice were treated with TMP or vehicle for 8 weeks. (a) Representative images of co‐immunofluorescence staining of p16^INK^ ^4a^ with LepR in longitudinal femoral sections. DAPI stains nuclei blue. Images of the upper panels are lower power with boxes outlining the area of higher power in bottom panels. (b–c) Quantitative analysis of the percentage of p16^INK^ ^4a+^‐expressing LepR^+^ cells (b) and Ki67‐expressing LepR^+^ cells (c) to all LepR^+^ cells. (d) Representative images of the flow cytometry sorting of CD45^−^LepR^+^ cells from bone marrow cells. The sorted cells were cultured with TMP or vehicle for 48 hr, and the p16^INK^ ^4A^ immunostaining (e), SA‐βGal staining (f) and BrdU incorporation (g) were performed. (e–g) Representative p16^INK^ ^4a^ (e), SA‐βGal (f) and Brdu (g) staining of LepR^+^ CD45^−^ cells treated with TMP or vehicle for 48 hr. (h–i) Quantitative analysis of the percentage of p16^INK^ ^4a^ (h), SA‐βGal (i) and Brdu (j) labelling cells to total sorted LepR^+^ CD45^−^ cells. (k–n) Quantitative RT‐PCR analysis of p16INK4a (k), p21 (l), Ki67 (m), p53 (n) expression in the sorted LepR^+^ CD45^−^ cells. Eight mice per group. Data are represented as mean ± SEM. MP, metaphysis. DP, diaphysis. *p < .05, **p < .01, NS, no significance as determined by two‐tailed Student's *t* tests

A previous study showed that LepR^+^ cells in bone marrow are the major subset of stem/progenitor cells contributing to bone formation and the maintenance of the haematopoietic cell niche in adults (Zhou et al., 2014). We investigated a large proportion of LepR^+^ cells displaying a senescence phenotype in aging mice, and TMP significantly decreased p16^+^‐expressing LepR^+^ cells and instead increased BrdU^+^‐labelled LepR^+^ cells (Figure 2a–c). To measure the direct effect of TMP on aging LepR^+^ bone marrow stem/progenitor cells (MSPCs), we sorted LepR^+^ cells from the bone marrow of aging mice using the marker LepR in combination with negative selection of CD45 (Figure 2d). We conducted exploratory SA‐βGal (Figure 2e), p16INK4a (Figure 2f) and BrdU staining (Figure 2g) in LepR^+^CD45^−^ MSPCs cultured with or without 50 μm TMP. p16INK4a^+^ and SA‐βGal^+^ LepR^+^ MSPCs significantly decreased after TMP treatment, while BrdU‐labelled LepR^+^ MSPCs markedly increased compared to vehicle control cells (Figure 2h–j). Moreover, TMP markedly decreased the mRNA levels of the senescent markers p16 and p21, while it increased the mRNA levels of the proliferative marker Ki67 (Figure 2k–m). However, the expression of p53, a tumour suppressor that controls the senescence response to tissue damage or cancer‐causing stress (Campisi, 2005), did not differ after TMP treatment (Figure 2n). These findings indicate the antisenescence and proliferative effects of TMP on aging LepR^+^ MSPCs.

### 2.3 The antisenescence effect of tetramethylpyrazine on LepR^+^ MSPCs is controlled by Ezh2‐H3K27me3

**Figure 3:**
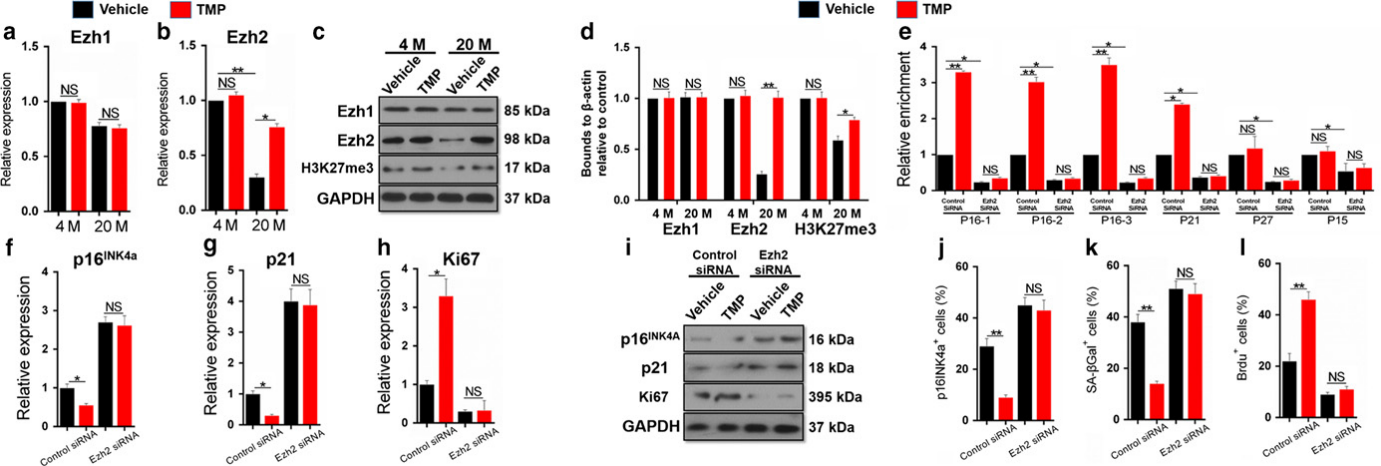
The antisenescence effect of tetramethylpyrazine in LepR^+^ MSPCs is controlled by Ezh2‐H3K27me3. FACs sorted LepR^+^ MSPCs from 4‐ and 20‐month‐old mice were treated with TMP or vehicle for 48 hr. (a–b) Quantitative RT‐PCR analysis of Ezh1 (K), and Ezh2 (b) expression in the sorted LepR^+^ MSPCs. (c–d) Western blot analysis and quantification of EZH1, EZH2 and H3K27me3 protein levels are shown. (e–l) FACs sorted LepR^+^ MSPCs from aging mice were transfected with EZH2 siRNA or control siRNA and were treated with TMP or vehicle for 48 hr. Chromatin immunoprecipitation (ChIP) with H3K27me3 antibody was performed (e). ChIP and input DNA were measured using real‐time PCR with specific primers targeting the promoter regions of the indicated genes (*n* = 3). Data are represented as mean ± SEM. *p < .05, **p < .01 as determined by ANOVA. Quantitative RT‐PCR analysis of p16INK4a (F), p21 (g) and Ki67 (h) expression in the sorted LepR^+^ MSPCs. Western blot analysis of p16INK4a, p21 and Ki67 protein levels is shown (i). Quantitative analysis of the percentage of p16^INK^ ^4a^ (j), SA‐βGal (k) and Brdu (l) labelling cells to total sorted LepR^+^ CD45^−^ cells. *n* = 5. Data are represented as mean ± SEM. *p < .05, **p < .01, NS, no significance as determined by two‐tailed Student's *t* tests

Increasing in vivo evidence suggests that dynamic chromatin modifications and local niche signals determine stem cell survival (Adam & Fuchs, 2016; Adam et al., 2015). The polycomb group (PcG) protein enhancer of zeste homologue 2 (Ezh2), which functions as a lysine *N*‐methyltransferase, mediates the repression of gene transcription through the trimethylation of histone H3 in lysine 27 (H3K27me3) to modulate progenitor cell senescence (Wei et al., 2011). We found that the mRNA and protein levels of Ezh2 were markedly decreased in LepR^+^ MSPCs of aging mice and that TMP treatment significantly induced Ezh2 expression and upregulated the protein levels of Ezh2 and H3K27me3 in LepR^+^ MSPCs (Figure 3a–d). We further confirmed these in vivo results by immunostaining of Ezh2 or H3K27me3 with LepR in aging mice treated with or without TMP for 8 weeks (Figure S4). The results showed that TMP significantly increased Ezh2^+^‐expressing LepR^+^ cells and H3K27me3^+^‐expressing LepR^+^ cells (Figure S4). However, the mRNA and protein levels of Ezh1, which is a homologue of Ezh2, remained unchanged during aging and after TMP treatment (Figure 3a–d). To measure whether TMP‐induced Ezh2 expression regulates cellular senescence by demethylating H3K27me3 in aging LepR^+^ MSPCs, chromatin immunoprecipitation (ChIP) assays were performed to assess changes in the histone methylation status at the promoter regions of the key genes involved in cell senescence and cell cycle arrest. We first knocked down Ezh2 by siRNA in LepR^+^ MSPCs (Figure S5) and found that in the control siRNA group, the promoter regions of the cell senescence inducer genes p16INK4a and p21CIP1 were enriched in H3K27me3 in TMP‐treated LepR^+^ MSPCs (Figure 3e). Interestingly, the enrichment of the promoter regions of p16INK4a and p21CIP1 was dramatically decreased after Ezh2 siRNA knock‐down, and TMP could not further increase their enrichment in LepR^+^ MSPCs (Figure 3e). We also tested the promoter of the cyclin‐dependent kinase (cdk) inhibitors p27^KIP1^ and p15^INK4b^; however, the degree of change in enrichment was not significant (Figure 3e). As a result, TMP significantly decreased the mRNA and protein levels of p16 and p21, while it increased the level of Ki67 (Figure 3f–i). However, knock‐down of Ezh2 markedly blocked the effect of TMP on p16, p21 and Ki67 (Figure 3f–i). Next, we performed SA‐βGal, p16INK4a and BrdU staining in TMP‐treated LepR^+^ MSPCs with or without Ezh2 knock‐down. We showed that TMP significantly decreased p16INK4a^+^ and SA‐βGal^+^ cells and increased BrdU^+^ cells as previously shown, while knock‐down of Ezh2 markedly blocked the antisenescence and proliferative effects of TMP (Figure 3j–l), which indicates the essential role of Ezh2 in modulating the TMP‐induced antisenescence and proliferative effects on aging LepR^+^ MSPCs.

### 2.4 Tetramethylpyrazine maintains HSCs in bone marrow and induces the expression of HSC maintenance genes in LepR^+^ MSPCs

**Figure 4:**
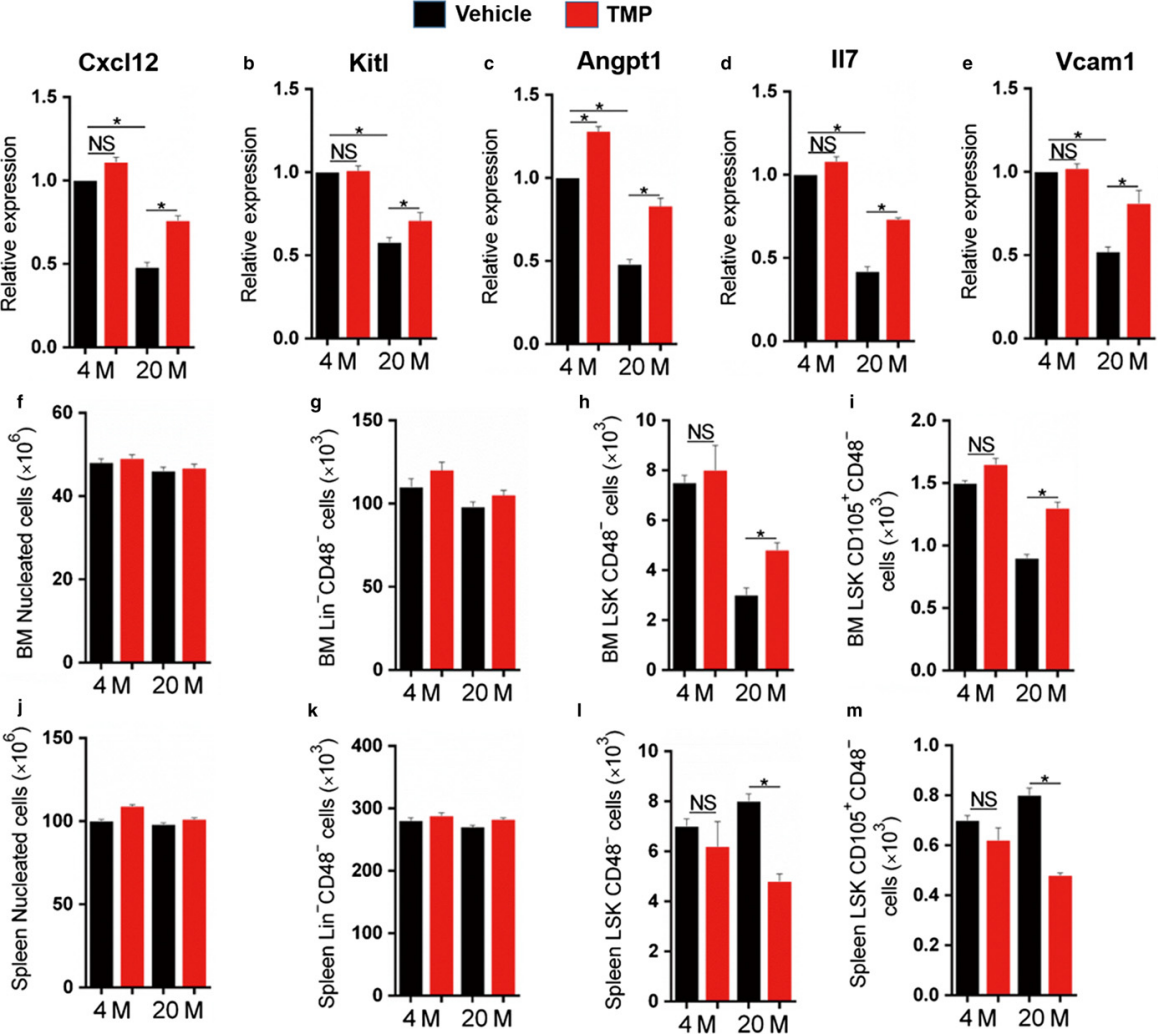
Tetramethylpyrazine maintains HSCs in the bone marrow and induces the expression of HSC maintenance genes in LepR^+^ MSPCs. FACs sorted LepR^+^ MSPCs from 4‐ and 20‐month‐old mice were treated with TMP or vehicle for 48 hr. (a–e) Quantitative RT‐PCR analysis of Cxcl12 (a), Kitl (b), Angpt1 (c), Il7 (d) and Ezh2 (b) expression in the sorted LepR^+^ MSPCs. (f–m) Measurement of bone marrow (BM) and spleen nucleated (f, j), Lin^−^ CD48^−^ (g, k), CD48^−^ LSK (h, l) and CD150^+^ CD48^−^ LSK (i, m) cells in 4‐month and 20‐month‐old male mice were treated with TMP or vehicle for 8 weeks. *n* = 5. Data are represented as mean ± SEM. *p < .05, **p < .01, NS, no significance as determined by two‐tailed Student's *t* tests

It is reported that LepR^+^ MSPCs is essential in maintaining the HSC niche (Zhou et al., 2014). To gain more insight into the regulatory effect of TMP on the bone marrow microenvironment and LepR^+^ MSPCs in aging mice, we sorted LepR^+^ MSPCs from 4‐ and 20‐month‐old mice treated with or without TMP and analysed the expression of genes that regulate HSC maintenance and attraction (Cxcl12, c‐kit ligand, angiopoietin‐1, interleukin‐7 and vascular cell adhesion molecule‐1). The expression of these genes significantly decreased in LepR^+^ MSPCs of aging mice relative to those of 4‐month‐old mice, and TMP potentially increased the levels of these genes in aging LepR^+^ MSPCs (Figure 4a–e). To further determine whether TMP maintains HSCs in bone marrow, we measured different lineages of haematopoietic cells in 4‐ and 20‐month‐old mice treated with or without TMP. Although bone marrow cellularity and Lin^−^CD48^−^ cell numbers were not significantly changed between different ages and treatment groups (Figure 4f,g), the more immature CD48^−^Lin^−^Sca‐1^+^c‐kit^+^ (LSK) cells (Figure 4h) and CD150^+^CD48^−^ LSK cells (Figure 4i) were markedly reduced in aging mice, while TMP treatment significantly increased and maintained the proportion of these cells (Figure 4h,i). Interestingly, the decreased number of LSK and CD150^+^CD48^−^LSK cells in bone marrow was associated with a proportional and selective increase in the number of these cells in the spleen of aging mice, and local delivery of TMP in bone marrow could significantly decrease the proportion of these cells in the spleen of aging mice (Figure 4j–m) without detectable differences in the cell cycle profile or apoptotic rates (Figure S6). These results indicate that the antisenescence effect of TMP on LepR^+^ MSPCs helps maintain HSCs and their niche in bone marrow and induces the mobilization of HSCs.

### 2.5 Tetramethylpyrazine induces H‐type vessel formation and creates an anti‐inflammatory and angiogenic environment in aging mice

**Figure 5:**
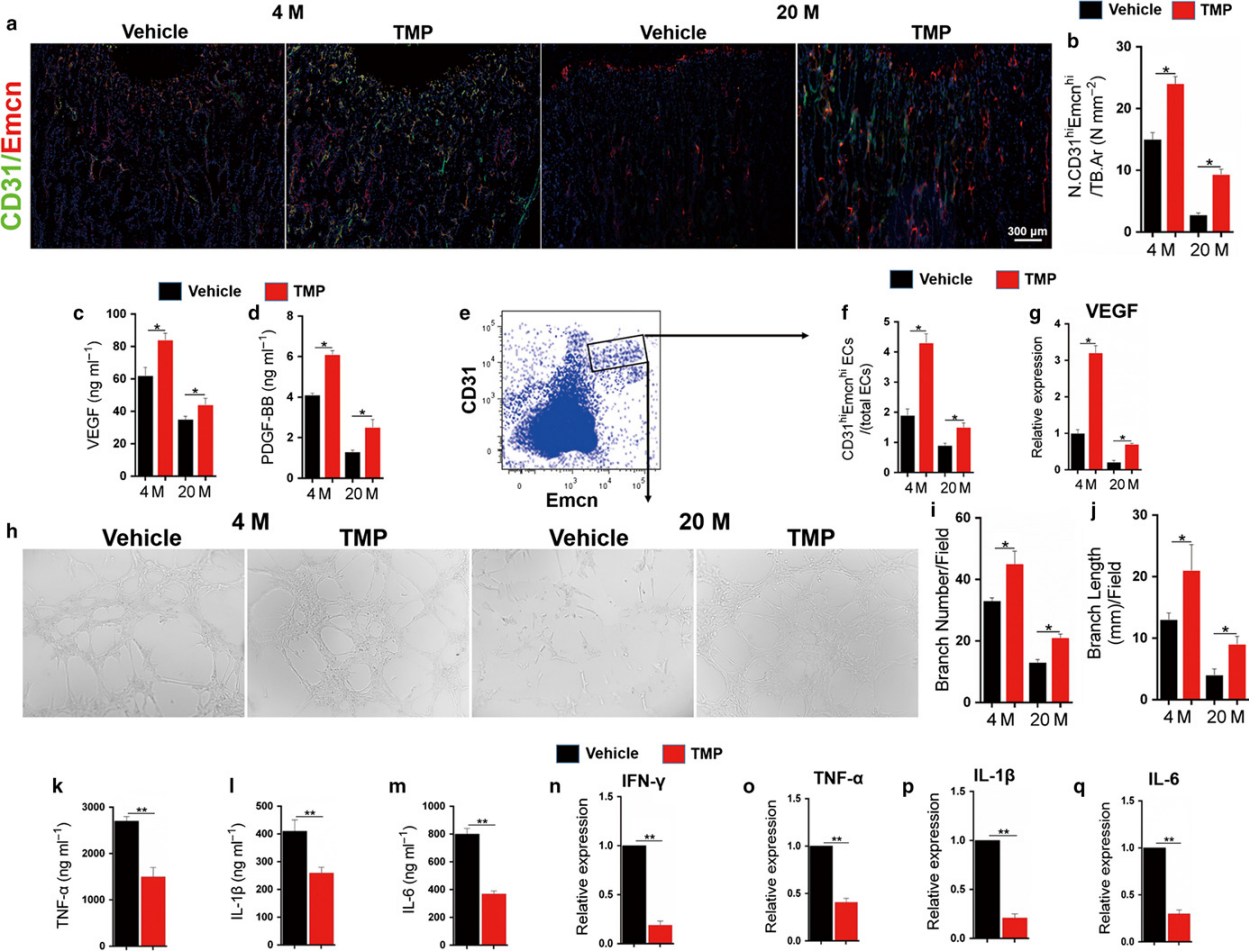
Tetramethylpyrazine induces H‐type vessel formation and creates an anti‐inflammatory and angiogenic environment in aging mice. 4‐ and 20‐month‐old male mice were treated with TMP or vehicle for 8 weeks. (a) Representative images of co‐immunofluorescence staining of CD31 with Emcn in longitudinal femoral sections. DAPI stains nuclei blue. (b) Quantitative analysis of the number of CD31^hi^Emcn^hi^ endothelial cells per mm^2^ tissue area (N. CD31^hi^Emcn^hi^/TB.Ar) in longitudinal femoral sections. (c–d) ELISA analysis for bone marrow (BM) VEGF (c) and PDGF‐BB (d). Eight mice per group. Data are represented as mean ± SEM. *p < .05 as determined by two‐tailed Student's *t* tests. (e) Representative images of FCM analysis and sorting of bone marrow CD31^hi^Emcn^hi^ endothelial cells. (F) Quantitative analysis of the percentage of bone marrow CD31^hi^Emcn^hi^ endothelial cell numbers to total endothelial cells in 4‐ and 20‐month‐old male mice treated with TMP or vehicle for 8 weeks. (g‐j) FCM sorted CD31^hi^Emcn^hi^ endothelial cells from 4‐ and 20‐month‐old male mice were treated with TMP or vehicle for 48 hr. Quantitative RT‐PCR analysis of VEGF expression in the sorted CD31^hi^Emcn^hi^ endothelial cells (g). Tube formation assays was performed (h) and branch numbers (i) and length (j) per field were analysed in sorted CD31^hi^Emcn^hi^ endothelial cells treated with TMP or vehicle. (k–m) ELISA analysis for bone marrow (BM) TNF‐α (k), IL‐1β (l) and IL‐6 (m) in 4‐ and 20‐month‐old male mice treated with TMP or vehicle for 8 weeks. (n–q) Quantitative RT‐PCR analysis of IFN‐γ (n), TNF‐α (o), IL‐1β (p) and IL‐6 (q) expression in bone marrow (BM) of 4‐ and 20‐month‐old male mice treated with TMP or vehicle for 8 weeks. *n* = 5. Data are represented as mean ± SEM. *p < .05, **p < .01, NS, no significance as determined by two‐tailed Student's *t* tests

H‐type (CD31^hi^Emcn^hi^) vessels have been reported to associate with a bioactive anti‐inflammatory microenvironment and new bone formation (Cui et al., 2016; Kusumbe et al., 2014). Whereas H‐type vessels markedly diminished in the bone marrow of aging mice, local injection of TMP not only significantly induced H‐type vessel formation (Figure 5a,b) but also increased the marrow VEGF and PDGF‐BB levels in both 4‐ and 20‐month‐old mice (Figure 5c,d), indicating the angiogenic effect of TMP on bone marrow. To further investigate the effect of TMP on angiogenesis and H‐type vessel formation, we performed FCM analysis in the bone marrow of 4‐ and 20‐month‐old mice treated with TMP or vehicle for 8 weeks (Figure 5e). We found that the number of CD31^hi^Emcn^hi^ endothelial cells was markedly reduced in aging mice, while TMP significantly increased their numbers (Figure 5f). Moreover, we sorted CD31^hi^Emcn^hi^ endothelial cells from 4‐ and 20‐month‐old mice and cultured them with 50 μm TMP or vehicle for 48 hr. Consistent with the angiogenic effect of TMP in vivo, the VEGF mRNA level in CD31^hi^Emcn^hi^ endothelial cells was markedly increased when cultured with TMP (Figure 5g). We also identified that TMP could markedly increase the tube formation and angiogenic sprouting of CD31^hi^Emcn^hi^ endothelial cells (Figure 5h–j), which further verified the effect of TMP on improving vascularization and the angiogenic microenvironment in the bone marrow of aging mice. Of note, we noticed that the local delivery of TMP in aging mice decreased the levels of anti‐inflammatory factors such as TNF‐α, IL‐1β and IL‐6 in bone marrow (Figure 5k–m) and that the mRNA levels of these factors were all also decreased by TMP treatment (Figure 5n–q). These findings indicate the essential role of tetramethylpyrazine in providing an anti‐inflammatory and angiogenic environment in aging mice.

### 2.6 AMPK‐mTOR‐Hif1α‐VEGF pathway is involved in the angiogenetic effect of tetramethylpyrazine on CD31^hi^Emcn^hi^ endothelial cells

**Figure 6:**
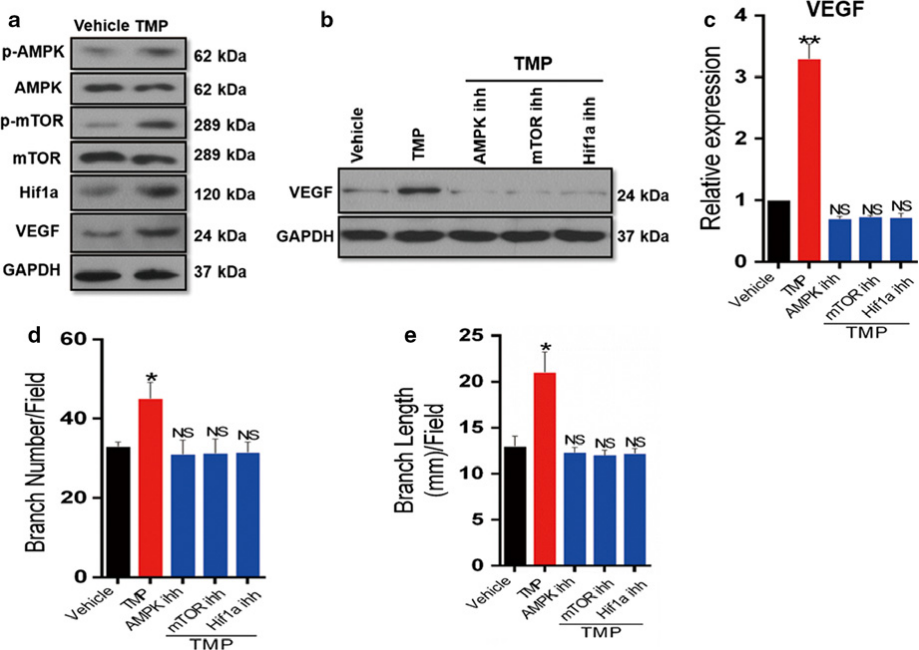
AMPK‐mTOR‐Hif1α‐VEGF pathway is involved in the angiogenetic effect of tetramethylpyrazine in CD31^hi^Emcn^hi^ endothelial cells. FACs sorted CD31^hi^Emcn^hi^ endothelial cells from 4‐ and 20‐month‐old mice were treated with TMP or vehicle for 48 hr. (a) Western blot analysis of total and phosphorylation of AMPK, mTOR, and Hif1α, VEGF protein levels are shown. (b–c) CD31^hi^Emcn^hi^ endothelial cells were treated with TMP or vehicle in the present or absent of various inhibitors as indicated. Western blot and quantitative RT‐PCR analysis of VEGF protein (b) and mRNA levels (c) are shown. (d–e) Tube formation assays were performed and branch numbers (d) and length (e) per field were analysed in sorted CD31^hi^Emcn^hi^ endothelial cells were treated with TMP or vehicle in the present or absent of various inhibitors as indicated. *n* = 5. Data are represented as mean ± SEM. *p < .05, **p < .01, NS, no significance as determined by ANOVA

To investigate the signalling pathway involved in TMP‐induced CD31^hi^Emcn^hi^ endothelial cells vessel formation, we sorted CD31^hi^Emcn^hi^ endothelial cells, and Western blot analysis showed that TMP induced the phosphorylation of AMPK and mTOR and upregulated Hif1α and VEGF protein levels (Figure 6a). Moreover, independent inhibition of AMPK, mTOR and Hif1α was sufficient to block the upregulation of VEGF protein levels induced by TMP (Figure 6b), which was further confirmed by RT‐PCR (Figure 6c), indicating that TMP induced VEGF via the AMPK‐mTOR‐Hif1α signalling pathway. Moreover, we showed that TMP could markedly increase the tube formation and angiogenic sprouting of CD31^hi^Emcn^hi^ endothelial cells, while independent inhibition of AMPK, mTOR and Hif1α was efficient in the inhibition of tube formation and angiogenic sprouting induced by TMP (Figure 6d,e). These results provide evidence that the AMPK‐mTOR‐Hif1α‐VEGF pathway is involved in the angiogenetic effects of tetramethylpyrazine on CD31^hi^Emcn^hi^ (H‐type) endothelial cells.

## 3 DISCUSSION

Senescent cell (SnC) accumulation in bone marrow with aging leads to aging‐related pathologies, and local ablation of SnCs attenuates several pathologic processes and extends a healthy lifespan (Finch, 2010; Jeon et al., 2017). There have been great efforts in understanding the mechanism of aging‐induced SnCs in bone marrow and the underlying potential treatment. In this study, we found that senescent LepR^+^ MSPCs accumulated in the bone marrow of aging mice with bone degeneration and that local delivery of TMP in bone marrow inhibited LepR^+^ MSPC senescence, which is essentially and epigenetically controlled by Ezh2‐H3K27me3. Moreover, TMP maintained the HSC niche and created an anti‐inflammatory and angiogenic environment in the bone marrow of aging mice.

Aging is associated with increased cellular senescence, which is characterized by profound chromatin/secretome changes and is associated with an abnormal microenvironment (Avrahami et al., 2015; Field & Adams, 2017). With dramatically increased bone marrow inflammatory factor levels, a sharply diminished blood supply and a markedly impaired local “stem cell niche” during aging, multiple cell types, especially “mesenchymal stromal/stem cells” in the bone microenvironment, become senescent. In this study, LepR^+^ cells in the bone marrow of aging mice displayed a senescent phenotype with a decreased proliferation ability. Zhou et al. (2014) first demonstrated that LepR^+^ cells in bone marrow were a major source of CFU‐F‐forming MSPCs in adults and formed bone, cartilage, and adipocytes in culture and upon transplantation in vivo. Considering these findings, we sorted LepR^+^ MSPCs from aging mice using flow cytometry and observed that LepR^+^ MSPCs also displayed a senescent phenotype with decreased BrdU labelling, which confirmed the increased cellular senescence of LepR^+^ MSPCs in aging bone marrow. The elimination of SnCs and inhibition of a certain SASP improves cardiovascular function (Roos et al., 2016), enhances insulin sensitivity (Xu, Palmer et al., 2015) and reduces frailty; (Xu, Tchkonia et al., 2015). Several recent studies have targeted local elimination of SnCs as a treatment for bone degenerative diseases, such as osteoarthritis and osteoporosis (Farr et al., 2017; Jeon et al., 2017). We previously determined that TMP could protect MSCs from glucocorticoid‐induced apoptosis and could be used for the prevention and treatment of GIOP (Wang et al., 2017). In this study, we further observed the local effect of TMP on the bone marrow of aging mice and, interestingly, found that the local delivery of TMP significantly decreased bone marrow SnCs and improved the metabolic microenvironment in aging mice (Figure 1). Moreover, we found that TMP could markedly inhibit the senescent phenotype and increase the proliferation of aging LepR^+^ MSPCs in vivo and in vitro (Figure 2). We further established the role of Ezh2‐H3K27me3 as a key epigenetic regulator in aging mice that controls the progression of LepR^+^ MSPC senescence during the local delivery of TMP. As a result, TMP inhibited the aging‐related loss of trabecular bone (which is metabolically modulated), while cortical bone (which is mechanically modulated) properties were not markedly changed. These findings indicate that the local elimination of senescent LepR MSPCs by TMP delays aging‐induced bone degeneration and improves the local metabolic microenvironment.

Bone marrow homeostasis is maintained in balance with the precise adjustment of various components, including HSC maintenance, the blood supply, anti‐inflammatory factors and proper metabolic responses. During aging, the bone marrow microenvironment is disrupted with multiple impaired physiologic processes. Adams et al. defined the H‐type vessel, which exhibits distinct molecular properties and is strongly positive for the endothelial cell surface markers CD31 and Emcn (CD31^hi^Emcn^hi^) as assessed by immunofluorescence staining and which is diminished during aging (Kusumbe et al., 2014). Additionally, the H‐type vessel modulates local microenvironments in the skeletal system, plays crucial roles in osteogenesis and provides niches for haematopoietic stem cells (Cui et al., 2016; Ramasamy, Kusumbe, Wang, & Adams, 2014). Interestingly, we sorted CD31^hi^Emcn^hi^ endothelial cells in the bone marrow of aging mice via flow cytometry and showed that TMP directly induced H‐type vessel formation in CD31^hi^Emcn^hi^ endothelial cells via the AMPK‐mTOR‐Hif1α‐VEGF pathway and promoted anti‐inflammatory factor upregulation in aging bone marrow. Ding, Saunders, Enikolopov, and Morrison (2012) showed that LepR^+^ cells are critical to the marrow environment and essential for HSC maintenance. Our work further found that TMP significantly increased HSC numbers and induced HSC maintenance in LepR^+^ MSPCs of aging mice. These findings indicate the crucial role of TMP in the antisenescence of MSPCs and in improving HSC maintenance. During aging, the local delivery of TMP not only inhibited LepR^+^ MSPCs directly via Ezh2‐H3k27me3 but also improved the whole marrow homeostasis and microenvironment, which will further ameliorate cellular senescence in aging bone marrow.

Of note, several studies have demonstrated that Ezh2 is an essential regulator of bone formation through modulating the osteogenic differentiation of MSCs (Chen et al., 2016; Hemming et al., 2016, 2017). Additionally, Li et al. (2017) found that Ezh2 also modulated the programmed senescence of MSCs during late puberty. He knocked out Ezh2^f/f^ in Nestin‐cre^ER^ and found that deletion of Ezh2 (Ezh2^−/−^) in early pubertal mice resulted in premature cellular senescence, depleted the MSPC pool, and impaired osteogenesis and osteoporosis in later life. Therefore, the function of Ezh2 in bone lies not only in promoting osteogenesis but also in inhibiting the senescence of MSCs. During aging, many MSCs are dysfunctional and undergo senescence, which leads to the disturbance of the bone marrow microenvironment, including interrupted HSC maintenance, inflammatory factor release and impaired angiogenesis and osteogenesis. Therefore, the priority of MSCs under senescence is “back to normal” and continuing their self‐renewal ability to maintain their own functions. Therefore, the local delivery of TMP in aging mice first recovered the function of the impaired MSCs by significantly inhibiting the senescent phenotype of LepR^+^ MSPCs by inducing EZH2 and simultaneously repaired the surrounding microenvironment by establishing an anti‐inflammatory and angiogenic environment.

In this study, we just began to understand that local elimination of senescent MSPCs in bone marrow is critical to aging‐related bone degenerative change and microenvironment disruption. Identification of the local treatment for cellular senescence and the underlying mechanism of the crosstalk between SnCs and niche cells in maintaining whole bone homeostasis remain interesting for further investigation, which will provide insight into extensive clinical studies in use of local treatment for bone degenerative and regenerative applications.

## 4 EXPERIMENTAL PROCEDURES

### 4.1 Animals and experimental procedures

Four‐month‐old young mice (weighing 30.84 ± 5.42 g) and 20‐month‐old aging C57BL/6J mice (weighing 47.8 ± 12.81 g) were obtained from the Experimental Animal Center at the Fourth Military Medical University in Xi'an, China, and were housed under specific pathogen‐free conditions (20°C, 12‐hr light/12‐hr dark cycles and 50%‐55% humidity) with free access to food and water. All mice from either 4‐month or 20‐month were randomly divided into different concentrations of TMP‐treated groups (0, 1, 10, 100, 1000 μg/kg) with eight mice per group. The mice were injected intramedullary with TMP or sesame oil (as a vehicle control), three times/week for 8 weeks. There was no significant difference in total body weight with same age among all the groups before the mice were sacrificed.

For Brdu administration, mice were given an intraperitoneal injection of 100 mg BrdU per kg body mass in Dulbecco's phosphate‐buffered saline (DPBS; Gibco) and were maintained on 1 mg/ml BrdU in the drinking water for 10 days before being sacrificed. Amber bottles containing BrdU water were changed every 1–3 days.

For intramedullary injection, TMP was delivered into the bone marrow cavity of mice from the medial side of the patellar tendon using 0.5‐mL syringes with 27‐gauge needles for 8 weeks as indicated above.

All experimental procedures in animals were approved by the Ethics in Animal Research Committee of the Fourth Military Medical University (Permission Code 20110405‐5).

### 4.2 Cell sorting and flow cytometry analysis

For flow cytometric sorting and analysis of CD45^−^LepR^+^ mesenchymal stem/progenitor cells (MSPCs) from femora, we dissected the femora free of soft tissues from 20‐ and 4‐month‐old mice. The bone was digested with a protease solution (2 mg/ml collagenase A and 2.5 mg/ml trypsin in phosphate‐buffered saline [PBS]) for 20 min to remove the periosteum and periosteal progenitors (step I). Then, the bones were cut into small pieces and digested in the protease solution for another 1 hr (step II). Cells within the supernatant were collected for flow cytometry. After the process of red blood cell lysis with commercial ammonium‐chloride‐potassium lysis buffer (Quality Biological, Inc., Gaithersburg, MD, USA), CD45^+^ cells were removed by CD45 MicroBeads using MACS cell separation system (Miltenyi Biotec, San Diego, CA, USA). Cells were then sorted according to side scatter and anti‐LepR‐biotin (BAF497; R&D Systems) after negative selection of CD45. For flow cytometric sorting of CD31^hi^Emcn^hi^ endothelial cells, cells were sorted using rat anti‐endomucin antibody (sc‐65495, Santa Cruz, 1:50) and goat anti‐CD31 antibody (AF3628; R&D Systems). For the labelling of HSCs, bone marrow cells from 4‐month‐old mice and 20‐month‐old mice treated with TMP or vehicle for 8 weeks were stained with biotinylated antibodies for haematopoietic lineages (detected with Pacific Orange‐conjugated streptavidin, Invitrogen, S32365), APC‐conjugated anti‐c‐kit (Biolegend, 105811), Pacific Blue‐conjugated anti‐Sca‐1 (Biolegend, 108119), PE‐conjugated anti‐CD150 (Biolegend, 115903) and FITC‐conjugated anti‐CD48 (Biolegend, 103402). FACS was performed using a 5‐laser BD FACS and FACSDiva (Becton Dickinson Biosciences, San Jose, CA, USA). Flow cytometric analyses were performed using Flowjo (Tree Star, Inc, Eugene, OR) and CellQuest software (Becton Dickinson Biosciences).

### 4.3 Isolation and labelling of HSCs

Bone marrow samples from aging congenic C57BL/6 mice were stained with biotinylated antibodies for haematopoietic lineages (detected with Pacific Orange‐conjugated streptavidin), APC‐conjugated anti‐c‐kit, Pacific Blue‐conjugated anti‐Sca‐1, PE‐conjugated anti‐CD150 and FITC‐conjugated anti‐CD48. CD150^+^CD48^−^LSK cells were sorted and stained with Vybrant DyD (Invitrogen).

### 4.4 Chromatin immunoprecipitation (ChIP) and antibodies

We first used Ezh2 siRNA assays (4390771; Thermo Fisher) to knock down Ezh2 in cells and performed CHIPs according to conditions of the manufacturer's EpiTect ChIP OneDay kit (Qiagen, Hilden, Germany) with ChIP‐grade antibodies to H3K27me3 (Qiagen). Briefly, we added formaldehyde to cells to cross‐link proteins to DNA, and the cells were lysed in 1.5‐mL lysis buffer (50 mm HEPES, pH 7.5, 140 mm NaCl; 1 mm EDTA; 1% Triton X‐100; 0.1% sodium deoxycholate; 0.1% sodium dodecyl sulphate). Cell lysates were sonicated at the set of 2 s on/15 s off for three rounds using a Bioruptor ultrasonic cell disruptor (Diagenode, Denville, NJ, USA) to shear genomic DNA to an average fragment size of 150 to 250 bp. Of the sample, 1% was removed for use as an input control. ChIP was performed following protocol provided by EpiTect ChIP OneDay kit (Qiagen) using antibodies towards H3K27Me3 (Qiagen). Anti‐RNA polymerase II and control IgG were used as positive and negative controls, respectively. After washing and de‐cross‐linking, the precipitated DNA was purified using a QIAquick PCR purification kit (Qiagen).

### 4.5 ChIP‐qPCR

ChIP‐qPCR was performed using SYBR Green PCR Master Mix and 7900 HT Fast Real‐Time PCR System (Applied Biosystems Corp., Foster City, CA, USA). Primers for p16, p21, P27 and P15 were used (see Table S1 for primer sequences). Absolute quantification was performed and enrichment expressed as a fraction of the whole‐cell extract control.

### 4.6 Preparing total RNA for quantitative reverse transcription PCR (qRT‐PCR)

Cells were sorted directly into Trizol. Total RNA was extracted according to the manufacturer's instructions (Invitrogen). Total RNA was subjected to reverse transcription and then qRT‐PCR using SYBR Green on a LightCycler 480 (Roche). Primers used in this study were listed in Table S2 for primer sequences.

For methods related to microcomputed tomography assessment, immunocytochemistry, immunofluorescence, cell culture, tube formation assay, Western blot and ELISA, see the Appendix S1.

### 4.7 Statistics and reproducibility

Data are presented as means ± standard errors of the mean. Unpaired, two‐tailed Student's *t* tests were used for comparisons between two groups. For multiple comparisons, one‐way analysis of variance (ANOVA) with Bonferroni post hoc test was applied. All data were normally distributed and had similar variation between groups. Statistical analysis was performed using sas version 9.3 software (SAS Institute, Inc., Cary, NC, USA). *p* < .05 was deemed significant.

## AUTHOR CONTRIBUTIONS

L.Y. and B.G. designed the experiments; B.G., X.S.L. and H.J. carried out most of the experiments; J.F., Q.J., C.C.J, X.L.X, D. W, W.G.L. and Y.Q.H. helped to collect the samples. C.Z. proofread the manuscript; L.Y. and Z.J.L supervised the experiments, analysed results and wrote the manuscript.

## CONFLICT OF INTEREST

No competing financial interests exist. No benefits in any form have been or will be received from a commercial party directly or indirectly by the authors of this article.

## References

[acel12741-bib-0001] Adam, R. C. , & Fuchs, E. (2016). The Yin and Yang of chromatin dynamics in stem cell fate selection. Trends in Genetics, 32, 89–100. 26689127 10.1016/j.tig.2015.11.002 PMC4733603

[acel12741-bib-0002] Adam, R. C. , Yang, H. , Rockowitz, S. , Larsen, S. B. , Nikolova, M. , Oristian, D. S. , … Fuchs, E. (2015). Pioneer factors govern super‐enhancer dynamics in stem cell plasticity and lineage choice. Nature, 521, 366–370. 25799994 10.1038/nature14289 PMC4482136

[acel12741-bib-0003] Alt, E. U. , Senst, C. , Murthy, S. N. , Slakey, D. P. , Dupin, C. L. , Chaffin, A. E. , … Izadpanah, R. (2012). Aging alters tissue resident mesenchymal stem cell properties. Stem Cell Research, 8, 215–225. 22265741 10.1016/j.scr.2011.11.002

[acel12741-bib-0004] Asada, N. , Kunisaki, Y. , Pierce, H. , Wang, Z. , Fernandez, N. F. , Birbrair, A. , … Frenette, P. S. (2017). Differential cytokine contributions of perivascular haematopoietic stem cell niches. Nature Cell Biology, 19, 214–223. 28218906 10.1038/ncb3475 PMC5467892

[acel12741-bib-0005] Avrahami, D. , Li, C. , Zhang, J. , Schug, J. , Avrahami, R. , Rao, S. , … Kaestner, K. H. (2015). Aging‐dependent demethylation of regulatory elements correlates with chromatin state and improved beta cell function. Cell Metabolism, 22, 619–632. 26321660 10.1016/j.cmet.2015.07.025 PMC4598285

[acel12741-bib-0006] Bonyadi, M. , Waldman, S. D. , Liu, D. , Aubin, J. E. , Grynpas, M. D. , & Stanford, W. L. (2003). Mesenchymal progenitor self‐renewal deficiency leads to age‐dependent osteoporosis in Sca‐1/Ly‐6A null mice. Proceedings of the National Academy of Sciences of the United States of America, 100, 5840–5845. 12732718 10.1073/pnas.1036475100 PMC156288

[acel12741-bib-0007] Cakouros, D. , Isenmann, S. , Cooper, L. , Zannettino, A. , Anderson, P. , Glackin, C. , & Gronthos, S. (2012). Twist‐1 induces Ezh2 recruitment regulating histone methylation along the Ink4A/Arf locus in mesenchymal stem cells. Molecular and Cellular Biology, 32, 1433–1441. 22290439 10.1128/MCB.06315-11 PMC3318575

[acel12741-bib-0008] Campisi, J. (2000). Cancer, aging and cellular senescence. In Vivo, 14, 183–188. 10757076

[acel12741-bib-0009] Campisi, J. (2005). Senescent cells, tumor suppression, and organismal aging: Good citizens, bad neighbors. Cell, 120, 513–522. 15734683 10.1016/j.cell.2005.02.003

[acel12741-bib-0010] Campisi, J. (2013). Aging, cellular senescence, and cancer. Annual Review of Physiology, 75, 685–705. 10.1146/annurev-physiol-030212-183653 PMC4166529 23140366

[acel12741-bib-0011] Chen, L. , Cheng, L. , Wei, X. , Yuan, Z. , Wu, Y. , Wang, S. , … Liu, H. (2017). Tetramethylpyrazine analogue CXC195 protects against dopaminergic neuronal apoptosis via activation of PI3K/Akt/GSK3beta signaling pathway in 6‐OHDA‐induced Parkinson's disease mice. Neurochemical Research, 42, 1141–1150. 28005221 10.1007/s11064-016-2148-x

[acel12741-bib-0012] Chen, Y. H. , Chung, C. C. , Liu, Y. C. , Yeh, S. P. , Hsu, J. L. , Hung, M. C. , … Li, L. Y. (2016). Enhancer of zeste homolog 2 and histone deacetylase 9c regulate age‐dependent mesenchymal stem cell differentiation into osteoblasts and adipocytes. Stem Cells, 34, 2183–2193. 27250566 10.1002/stem.2400

[acel12741-bib-0013] Chen, L. , Wei, X. , Hou, Y. , Liu, X. , Li, S. , Sun, B. , … Liu, H. (2014). Tetramethylpyrazine analogue CXC195 protects against cerebral ischemia/reperfusion‐induced apoptosis through PI3K/Akt/GSK3beta pathway in rats. Neurochemistry International, 66, 27–32. 24462584 10.1016/j.neuint.2014.01.006

[acel12741-bib-0014] Coppe, J. P. , Patil, C. K. , Rodier, F. , Sun, Y. , Munoz, D. P. , Goldstein, J. , … Campisi, J. (2008). Senescence‐associated secretory phenotypes reveal cell‐nonautonomous functions of oncogenic RAS and the p53 tumor suppressor. PLoS Biology, 6, 2853–2868. 19053174 10.1371/journal.pbio.0060301 PMC2592359

[acel12741-bib-0015] Cui, Z. , Crane, J. , Xie, H. , Jin, X. , Zhen, G. , Li, C. , … Cao, X. (2016). Halofuginone attenuates osteoarthritis by inhibition of TGF‐beta activity and H‐type vessel formation in subchondral bone. Annals of the Rheumatic Diseases, 75, 1714–1721. 26470720 10.1136/annrheumdis-2015-207923 PMC5013081

[acel12741-bib-0016] Ding, L. , Saunders, T. L. , Enikolopov, G. , & Morrison, S. J. (2012). Endothelial and perivascular cells maintain haematopoietic stem cells. Nature, 481, 457–462. 22281595 10.1038/nature10783 PMC3270376

[acel12741-bib-0017] Farr, J. N. , Xu, M. , Weivoda, M. M. , Monroe, D. G. , Fraser, D. G. , Onken, J. L. , … Khosla, S. (2017). Targeting cellular senescence prevents age‐related bone loss in mice. Nature Medicine, 23, 1072–1079. 10.1038/nm.4385 PMC5657592 28825716

[acel12741-bib-0018] Fehrer, C. , Laschober, G. , & Lepperdinger, G. (2006). Aging of murine mesenchymal stem cells. Annals of the New York Academy of Sciences, 1067, 235–242. 16803992 10.1196/annals.1354.030

[acel12741-bib-0019] Field, A. E. , & Adams, P. D. (2017). Targeting chromatin aging – The epigenetic impact of longevity‐associated interventions. Experimental Gerontology, 94, 29–33. 27986499 10.1016/j.exger.2016.12.010

[acel12741-bib-0020] Finch, C. E. (2010). Evolution in health and medicine Sackler colloquium: Evolution of the human lifespan and diseases of aging: Roles of infection, inflammation, and nutrition. Proceedings of the National Academy of Sciences of the United States of America, 107(Suppl. 1), 1718–1724. 19966301 10.1073/pnas.0909606106 PMC2868286

[acel12741-bib-0021] Gong, X. , Ivanov, V. N. , Davidson, M. M. , & Hei, T. K. (2015). Tetramethylpyrazine (TMP) protects against sodium arsenite‐induced nephrotoxicity by suppressing ROS production, mitochondrial dysfunction, pro‐inflammatory signaling pathways and programed cell death. Archives of Toxicology, 89, 1057–1070. 24961358 10.1007/s00204-014-1302-y PMC4377316

[acel12741-bib-0022] Hemming, S. , Cakouros, D. , Codrington, J. , Vandyke, K. , Arthur, A. , Zannettino, A. , & Gronthos, S. (2017). EZH2 deletion in early mesenchyme compromises postnatal bone microarchitecture and structural integrity and accelerates remodeling. FASEB Journal, 31, 1011–1027. 27934660 10.1096/fj.201600748R

[acel12741-bib-0023] Hemming, S. , Cakouros, D. , Vandyke, K. , Davis, M. J. , Zannettino, A. C. , & Gronthos, S. (2016). Identification of novel EZH2 targets regulating osteogenic differentiation in mesenchymal stem cells. Stem Cells and Development, 25, 909–921. 27168161 10.1089/scd.2015.0384 PMC4928132

[acel12741-bib-0024] Iyer, S. , Brooks, R. , Gumbleton, M. , & Kerr, W. G. (2015). SHIP1‐expressing mesenchymal stem cells regulate hematopoietic stem cell homeostasis and lineage commitment during aging. Stem Cells and Development, 24, 1073–1081. 25525673 10.1089/scd.2014.0501 PMC4403265

[acel12741-bib-0025] Jeon, O. H. , Kim, C. , Laberge, R. M. , Demaria, M. , Rathod, S. , Vasserot, A. P. , … Elisseeff, J. H. (2017). Local clearance of senescent cells attenuates the development of post‐traumatic osteoarthritis and creates a pro‐regenerative environment. Nature Medicine, 23, 775–781. 10.1038/nm.4324 PMC5785239 28436958

[acel12741-bib-0026] Kfoury, Y. , & Scadden, D. T. (2015). Mesenchymal cell contributions to the stem cell niche. Cell Stem Cell, 16, 239–253. 25748931 10.1016/j.stem.2015.02.019

[acel12741-bib-0027] Kusumbe, A. P. , Ramasamy, S. K. , & Adams, R. H. (2014). Coupling of angiogenesis and osteogenesis by a specific vessel subtype in bone. Nature, 507, 323–328. 24646994 10.1038/nature13145 PMC4943525

[acel12741-bib-0028] Lepperdinger, G. (2011). Inflammation and mesenchymal stem cell aging. Current Opinion in Immunology, 23, 518–524. 21703839 10.1016/j.coi.2011.05.007 PMC3167021

[acel12741-bib-0029] Li, C. , Chai, Y. , Wang, L. , Gao, B. , Chen, H. , Gao, P. , … Wan, M. (2017). Programmed cell senescence in skeleton during late puberty. Nature Communications, 8, 1312. 10.1038/s41467-017-01509-0 PMC5670205 29101351

[acel12741-bib-0030] Margueron, R. , & Reinberg, D. (2011). The polycomb complex PRC2 and its mark in life. Nature, 469, 343–349. 21248841 10.1038/nature09784 PMC3760771

[acel12741-bib-0031] Mendelson, A. , & Frenette, P. S. (2014). Hematopoietic stem cell niche maintenance during homeostasis and regeneration. Nature Medicine, 20, 833–846. 10.1038/nm.3647 PMC4459580 25100529

[acel12741-bib-0032] Nelson, G. , Wordsworth, J. , Wang, C. , Jurk, D. , Lawless, C. , Martin‐Ruiz, C. , & von Zglinicki, T. (2012). A senescent cell bystander effect: Senescence‐induced senescence. Aging Cell, 11, 345–349. 22321662 10.1111/j.1474-9726.2012.00795.x PMC3488292

[acel12741-bib-0033] Ramasamy, S. K. , Kusumbe, A. P. , Wang, L. , & Adams, R. H. (2014). Endothelial Notch activity promotes angiogenesis and osteogenesis in bone. Nature, 507, 376–380. 24647000 10.1038/nature13146 PMC4943529

[acel12741-bib-0034] Rodier, F. , & Campisi, J. (2011). Four faces of cellular senescence. Journal of Cell Biology, 192, 547–556. 21321098 10.1083/jcb.201009094 PMC3044123

[acel12741-bib-0035] Roos, C. M. , Zhang, B. , Palmer, A. K. , Ogrodnik, M. B. , Pirtskhalava, T. , Thalji, N. M. , … Miller, J. D. (2016). Chronic senolytic treatment alleviates established vasomotor dysfunction in aged or atherosclerotic mice. Aging Cell, 15, 973–977. 26864908 10.1111/acel.12458 PMC5013022

[acel12741-bib-0036] Serrano, M. , Lin, A. W. , McCurrach, M. E. , Beach, D. , & Lowe, S. W. (1997). Oncogenic ras provokes premature cell senescence associated with accumulation of p53 and p16INK4a. Cell, 88, 593–602. 9054499 10.1016/s0092-8674(00)81902-9

[acel12741-bib-0037] Stenderup, K. , Justesen, J. , Clausen, C. , & Kassem, M. (2003). Aging is associated with decreased maximal life span and accelerated senescence of bone marrow stromal cells. Bone, 33, 919–926. 14678851 10.1016/j.bone.2003.07.005

[acel12741-bib-0038] Sun, Y. , Yu, P. , Zhang, G. , Wang, L. , Zhong, H. , Zhai, Z. , … Wang, Y. (2012). Therapeutic effects of tetramethylpyrazine nitrone in rat ischemic stroke models. Journal of Neuroscience Research, 90, 1662–1669. 22431378 10.1002/jnr.23034

[acel12741-bib-0039] Wang, L. , Zhang, H. Y. , Gao, B. , Shi, J. , Huang, Q. , Han, Y. H. , … Luo, Z. J. (2017). Tetramethylpyrazine protects against glucocorticoid‐induced apoptosis by promoting autophagy in mesenchymal stem cells and improves bone mass in glucocorticoid‐induced osteoporosis rats. Stem Cells and Development, 26, 419–430. 27917698 10.1089/scd.2016.0233

[acel12741-bib-0040] Wei, Y. , Chen, Y. H. , Li, L. Y. , Lang, J. , Yeh, S. P. , Shi, B. , … Hung, M. C. (2011). CDK1‐dependent phosphorylation of EZH2 suppresses methylation of H3K27 and promotes osteogenic differentiation of human mesenchymal stem cells. Nature Cell Biology, 13, 87–94. 21131960 10.1038/ncb2139 PMC3076036

[acel12741-bib-0041] Xu, M. , Palmer, A. K. , Ding, H. , Weivoda, M. M. , Pirtskhalava, T. , White, T. A. , … Kirkland, J. L. (2015). Targeting senescent cells enhances adipogenesis and metabolic function in old age. Elife, 4, e12997. 26687007 10.7554/eLife.12997 PMC4758946

[acel12741-bib-0042] Xu, M. , Tchkonia, T. , Ding, H. , Ogrodnik, M. , Lubbers, E. R. , Pirtskhalava, T. , … Kirkland, J. L. (2015). JAK inhibition alleviates the cellular senescence‐associated secretory phenotype and frailty in old age. Proceedings of the National Academy of Sciences of the United States of America, 112, E6301–E6310. 26578790 10.1073/pnas.1515386112 PMC4655580

[acel12741-bib-0043] Yu, N. , Zhang, Z. , Chen, P. , Zhong, Y. , Cai, X. , Hu, H. , … Zhuang, J. (2015). Tetramethylpyrazine (TMP), an active ingredient of chinese herb medicine chuanxiong, attenuates the degeneration of trabecular meshwork through SDF‐1/CXCR4 axis. PLoS ONE, 10, e133055. 10.1371/journal.pone.0133055 PMC4537220 26275042

[acel12741-bib-0044] Zang, L. , Hao, H. , Liu, J. , Li, Y. , Han, W. , & Mu, Y. (2017). Mesenchymal stem cell therapy in type 2 diabetes mellitus. Diabetology & Metabolic Syndrome, 9, 36. 28515792 10.1186/s13098-017-0233-1 PMC5433043

[acel12741-bib-0045] Zhou, S. , Greenberger, J. S. , Epperly, M. W. , Goff, J. P. , Adler, C. , Leboff, M. S. , & Glowacki, J. (2008). Age‐related intrinsic changes in human bone‐marrow‐derived mesenchymal stem cells and their differentiation to osteoblasts. Aging Cell, 7, 335–343. 18248663 10.1111/j.1474-9726.2008.00377.x PMC2398731

[acel12741-bib-0046] Zhou, B. O. , Yue, R. , Murphy, M. M. , Peyer, J. G. , & Morrison, S. J. (2014). Leptin‐receptor‐expressing mesenchymal stromal cells represent the main source of bone formed by adult bone marrow. Cell Stem Cell, 15, 154–168. 24953181 10.1016/j.stem.2014.06.008 PMC4127103

