# Supplementary figures and images for: Local delivery of tetramethylpyrazine eliminates the senescent phenotype of bone marrow mesenchymal stromal cells and creates an anti‐inflammatory and angiogenic environment in aging mice
Source: Aging Cell. 2018 Feb 28;17(3):e12741. doi: 10.1111/acel.12741 (PMC5946084; doi:10.1111/acel.12741)

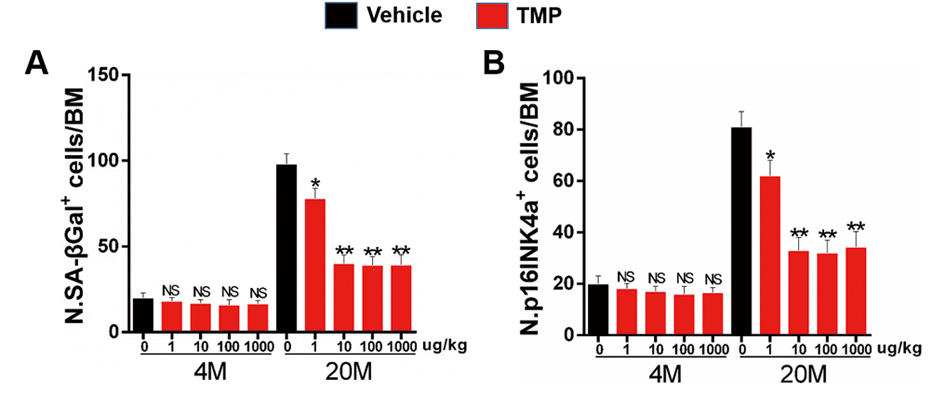

Supplement: Supplementary file 1 [file ACEL-17-e12741-s001.tif]

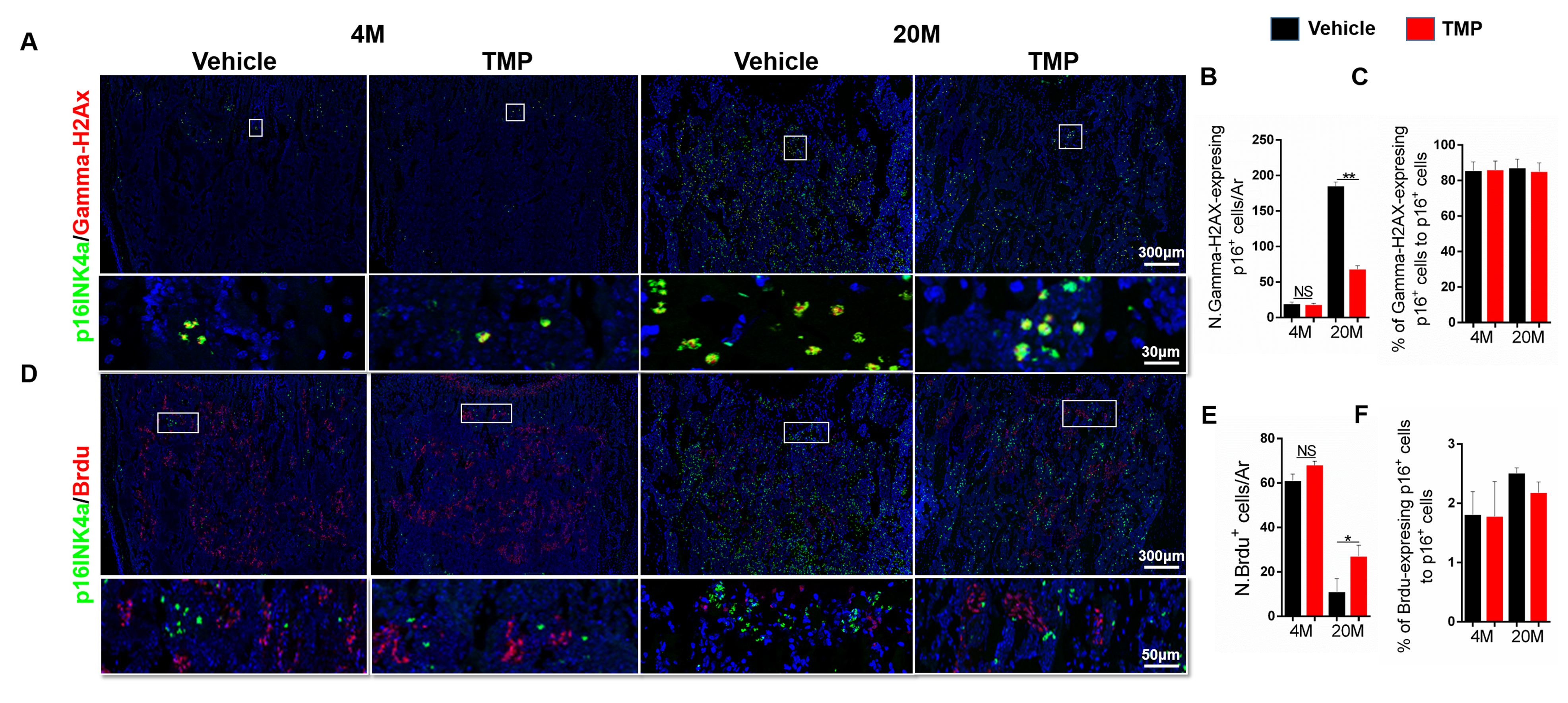

Supplement: Supplementary file 2 [file ACEL-17-e12741-s002.tif]

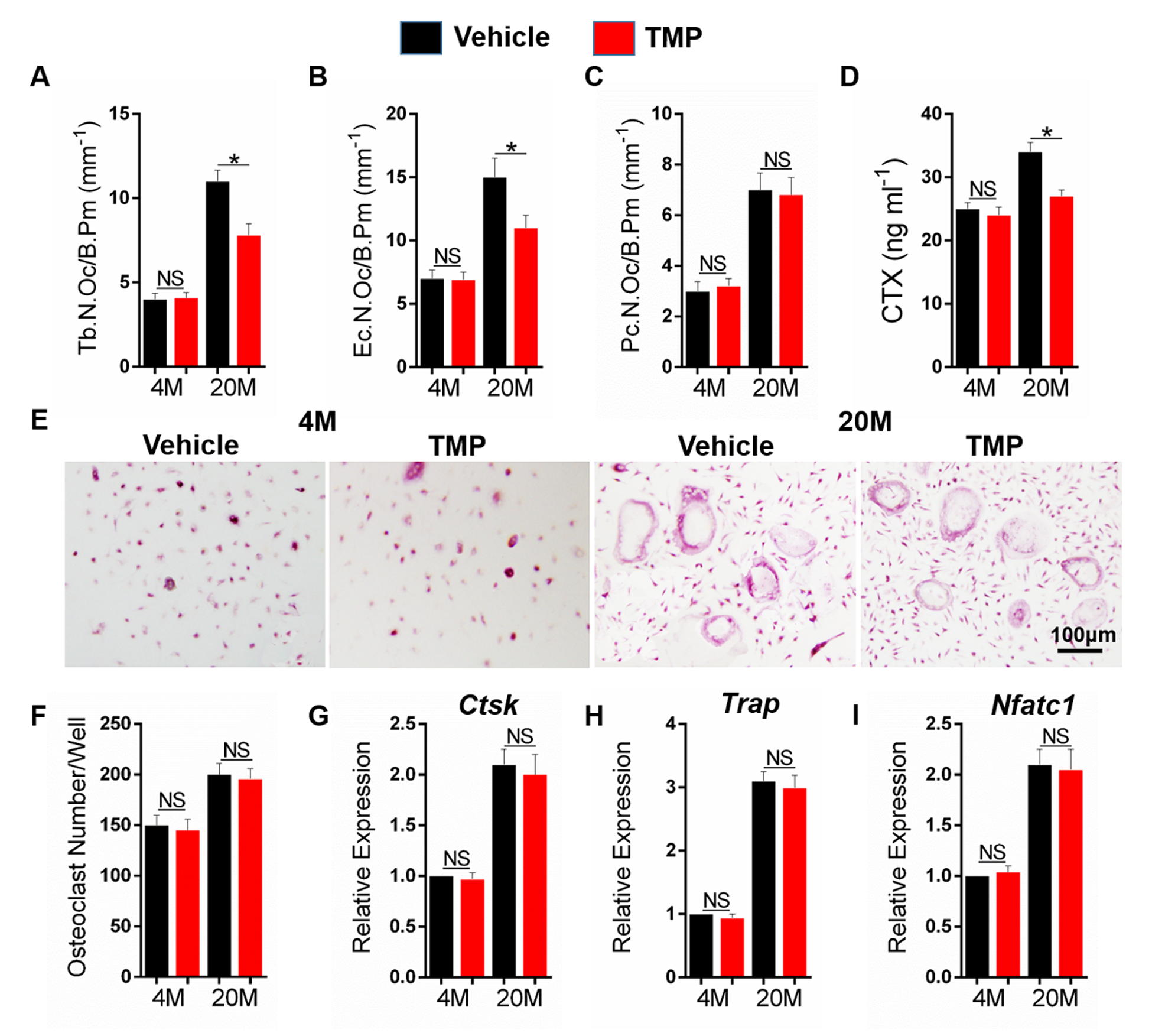

Supplement: Supplementary file 3 [file ACEL-17-e12741-s003.tif]

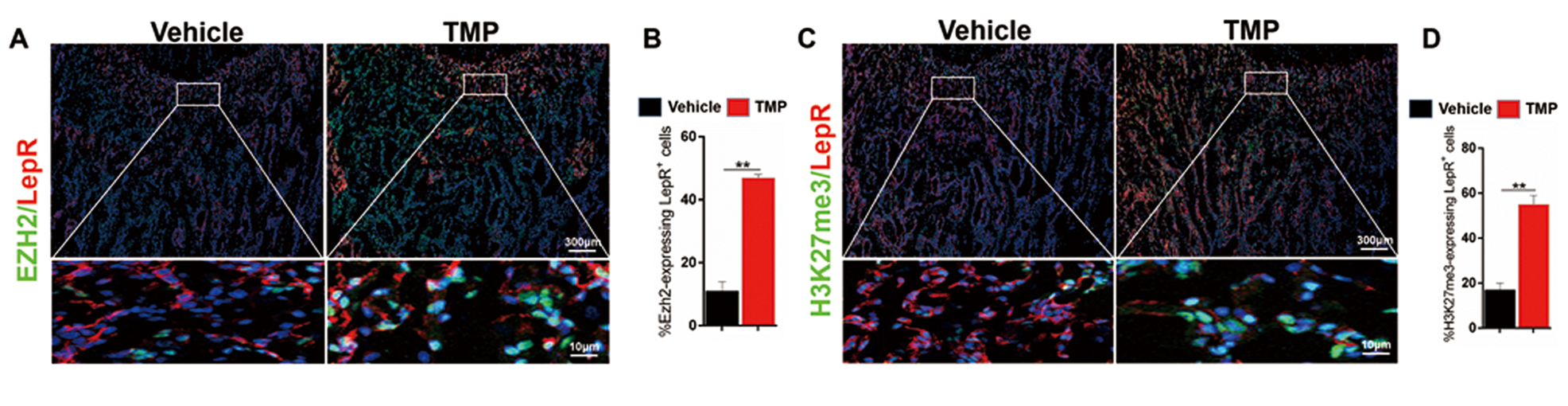

Supplement: Supplementary file 4 [file ACEL-17-e12741-s004.tif]

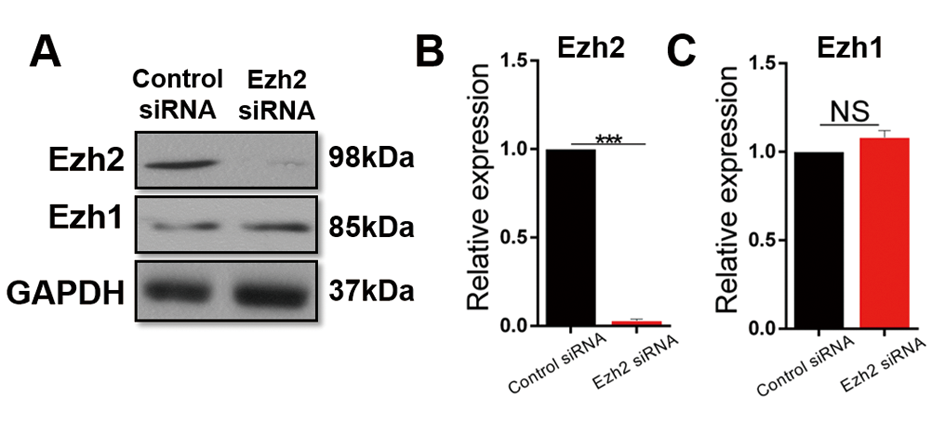

Supplement: Supplementary file 5 [file ACEL-17-e12741-s005.tif]

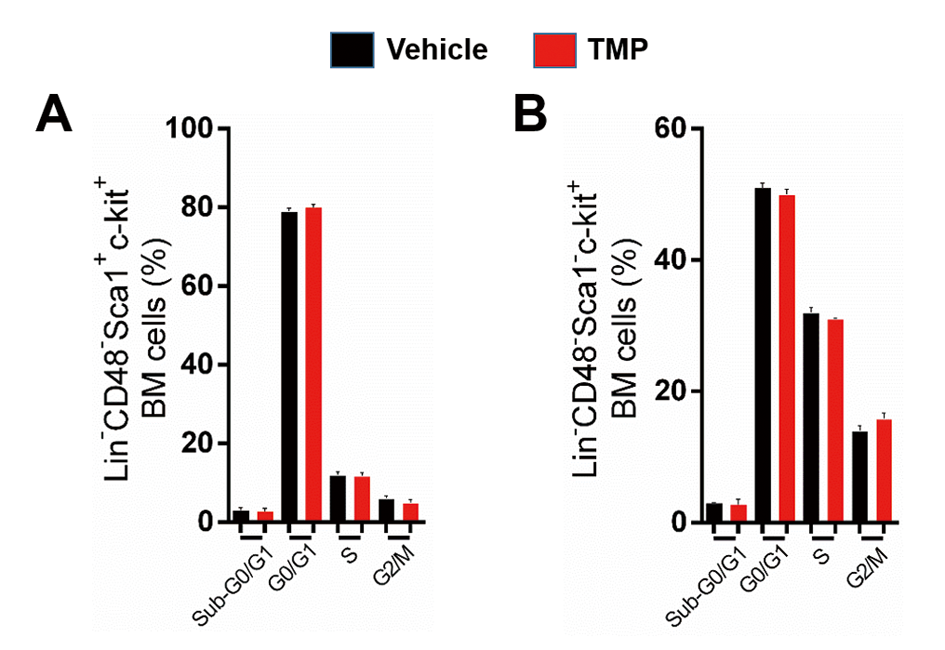

Supplement: Supplementary file 6 [file ACEL-17-e12741-s006.tif]
