# Supplementary material for: Local delivery of tetramethylpyrazine eliminates the senescent phenotype of bone marrow mesenchymal stromal cells and creates an anti‐inflammatory and angiogenic environment in aging mice
Source: Aging Cell. 2018 Feb 28;17(3):e12741. doi: 10.1111/acel.12741 (PMC5946084; doi:10.1111/acel.12741)
Supplement: Supplementary file 7 [file ACEL-17-e12741-s007.docx]

**Supplementary Table 1. Primers used for ChIP-PCR**

| **Primers** | **Forward** | **Reverse** |
| --- | --- | --- |
| p15^INK4b^ | CCGCCTAGAGATCGAACTAGCC | CATTTGTGCATAGGAGATCAGG |
| p16^INK4a^-1 | TCCGATCCTTTAGCGCTGTT | CCCGGACTACAGAAGAGATG |
| p16^INK4a^-2 | AGGGGTGTTCAATTCATGCTAT | ACACTCTGCTCCTGACCTGG |
| p16^INK4a^-3 | GGAGCCACCCATTAAACTAACT | CAAAAATAAGACACTGAAAACTCG |
| p21^CIP1^ | CACAGTTGGTCAGGGACAGA | CAGGACCAACCCACTCCTT |
| p27^KIP1^ | CTGGCTCTGCTCCATTTGAC | GGCTCCCGTTAGACACTCTC |
