# Supplementary material for: Local delivery of tetramethylpyrazine eliminates the senescent phenotype of bone marrow mesenchymal stromal cells and creates an anti‐inflammatory and angiogenic environment in aging mice
Source: Aging Cell. 2018 Feb 28;17(3):e12741. doi: 10.1111/acel.12741 (PMC5946084; doi:10.1111/acel.12741)
Supplement: Supplementary file 8 [file ACEL-17-e12741-s008.docx]

**Supplementary Table 2. Primers used for quantitative real-time PCR**

| **Primers** | **Forward** | **Reverse** |
| --- | --- | --- |
| p16^INK4a^ | GAAAGAGTTCGGGGCGTTG | GAGAGCCATCTGGAGCAGCAT |
| p21^CIP1^ | AGAAGGTACTTACGGTGTGGT | GAGAGATTTCCCGAATTGCAGT |
| p53 | ATCGCCTTCGACATCATCGC | CCCCATGCGTACTCCATGAG |
| Ki67 | ACCGTGGAGTAGTTTATCTGGG | TGTTTCCAGTCCGCTTACTTCT |
| Ezh1 | CCAGACTGCCAGAATCGCTTT | CAGGTGCTTTTTGAGGCCA |
| Ezh2 | AGTGACTTGGATTTTCCAGCAC | AATTCTGTTGTAAGGGCGACC |
| Cxcl12 | CGCCAAGGTCGTCGCCG | TTGGCTCTGGCGATGTGGC |
| Kitl | CCCTGAAGACTCGGGCCTA | CAATTACAAGCGAAATGAGAGCC |
| Angpt1 | CTCGTCAGACATTCATCATCCAG | CACCTTCTTTAGTGCAAAGGCT |
| Il7 | GTGCTGCTCGCAAGTTGAAG | AGTTCACCAGTGTTTGTGTGC |
| Vcam1 | GACCTGTTCCAGCGAGGGTCTA | CTTCCATCCTCATAGCAATTAAGGTG |
| Vegf | GGAGATCCTTCGAGGAGCACTT | GGCGATTTAGCAGCAGATATAAGAA |
| IFN-γ | AGCGGCTGACTGAACTCAGATTGTAG | GTCACAGTTTTCAGCTGTATAGGG |
| TNF-α | GGCAGGTCTACTTTGGAGTCATTGC | ACATTCGAGGCTCCAGTGAATTCGG |
| Il-1β | TTGACGGACCCCAAAAGATG | AGAAGGTGCTCATGTCCTCA |
| Il-6 | GTTCTCTGGGAAATCGTGGA | TGTACTC CAGGTAGCTATGG- |
| Trap | CGACCATTGTTAGCCACATACG | TCGTCCTGAAGATACTGCAGGTT |
| Nfatc1 | CAAGTCTCACCACAGGGCTCACTA | TCAGCCGTCCCAATGAACAG |
| Ctsk | CACCCAGTGGGAGCTATGGAA | GCCTCCAGGTTATGGGCAGA |
| β-actin | CATCCGTAAAGACCTCTATGCCAAC | ATGGAGCCACCGATCCACA |
