# Supplementary material for: Local delivery of tetramethylpyrazine eliminates the senescent phenotype of bone marrow mesenchymal stromal cells and creates an anti‐inflammatory and angiogenic environment in aging mice
Source: Aging Cell. 2018 Feb 28;17(3):e12741. doi: 10.1111/acel.12741 (PMC5946084; doi:10.1111/acel.12741)
Supplement: Supplementary file 9 [file ACEL-17-e12741-s009.docx]

**Supplemental experimental procedures**

**Microcomputed tomography assessment**

The distal femora were scanned using explore Locus SP Pre-Clinical Specimen micro-computed tomography (CT;GE Corp., Fairfield, CT) with an 8mm resolution, a 50 kV tube voltage, and a 0.1mA tube current. Reconstruction and three-dimensional (3D) quantitative analyses were determined using the software provided by a desktop micro-CT system (GE Corp.). Trabecular bone region of interest (ROI) consisted of about 120 slices was drawn starting from about 0.1 mm distal to the growth plate, constituting 0.70 mm in length. The trabecular bone was segmented from the bone marrow and analyzed to determine the trabecular bone volume fraction (BV/TV), trabecular thickness (Tb. Th). Diaphyseal cortical bone ROI was drawn starting from 20% of femoral length proximal to distal epiphyseal growth plate and extended proximally for a total of 10% of femoral length. We analyzed the cortical bone in order to determine the cortical thickness (Ct. Th) and periosteal perimeter (Ps. Pm).

**Immunocytochemistry, immunofluorescence and histomorphometry.**

At the time of euthanasia, mice femora were dissected and fixed in 10% buffered formalin for 20 h, decalcified in 10% EDTA (pH 7.4) (Amresco) for 2 d and embedded in paraffin or optimal cutting temperature compound (Sakura Finetek). We processed 4-mm-thick coronal (longitudinally) oriented sections of bone including the metaphysis and diaphysis for SA-βGal and p16INK4a staining. Longitudinally oriented 10-μm-thick sections of bone were processed for immunofluorescence staining. Briefly, we incubated the sections with primary antibodies to p16Ink4A (Abcam, ab189034, 1:100), LepR (R&D Systems, BAF497, 1:200), Brdu (Abcam, ab6326, 1:200), Gamma-H2AX (ab26350, abcam, 1:200), endomucin (sc-65495, Santa Cruz, diluted 1:100), pecam1(CD31) conjugated to Alexa Fluor 488 (R&D Systems, FAB3628G, 1:100), Ezh2 (Cell Signaling, 5206S,1:200), Histone H3K27me3 (Cell Signaling, 9733S, 1:300), followed by incubation with FITC, or Cy3-conjugated secondary antibodies (Jackson ImmunoResearch), or goat anti-rabbit, goat anti-mouse, donkey anti-goat (Abcam, 1:100) secondary antibodies. Nuclei were counterstained with DAPI (Sigma). We counted the numbers of the cells using a FluoView FV1000 confocal laser scanning microscope (Olympus Corporation, Tokyo, Japan).

**Cell culture and treatment**

Isolation and primary culture of MSPCs were performed based on flow-cytometry described above. Briefly, the sorted MSPCs were seeded into 75 cm^2^ culture flasks with a-minimum essential medium (Thermo Fisher Scientific, Waltham, MA), containing 10% fetal bovine serum and 1% penicillin-streptomycin (all from Gibco Life Technologies, Carlsbad, CA) under conditions of 5% CO_2_ and 37°C. The medium was changed every 2-3 days to remove non-adherent cells. When the adherent cells were confluent, they were detached using 0.25% Trypsin ethylenediaminetetraacetic acid (EDTA; Gibco Life Technologies) and passaged at a ratio of 1:2. MSPCs were incubated using a regular culture medium, which contained different concentrations of TMP (0, or 50 mM) for 48 h. The doses of TMP were chosen based on previous studies (20, 21). To measure the effects and mechanisms of TMP-induced angiogenesis, 10 nM of the mTOR inhibitor rapamycin (Cell Signaling Technology, Danvers, MA), 10 mM of the AMPK inhibitor compound C (Sigma-Aldrich) and 10 μM of Hif1a inhibitor 3-(2-(4-Adamantan-1-yl-phenoxy)-acetylamino)-4-hydroxybenzoic acid methylester (Santa Cruz, sc-205346) were added.

**Tube formation assay**

Sorted endothelial cells were grown in the EC medium (ScienCell Research Laboratories, Carlsbad, CA, USA) containing 5% FBS and EC growth supplement for 3-5 passages. When the cells reached 80% confluence, endothelial cells were incubated with conditioned medium for angiogenesis for 48 h and then trypsinized. The cells (1x 10^5^) were seeded in a 48-well dish pre-coated with 150 ml Matrigel synthetic basement membrane (BD Biosciences, San Jose, CA, USA) and were incubated for 6 h at 37°C. The tubes formed by sorted endothelial cells were observed and photographed in 5 randomly selected microscopic fields to determine the number and length of the branches. The quantification of the tubes was performed by using Image-Pro Plus 6.0 (Media Cybernetics, Rockville, MD, USA).

**Fibrin bead assay**

The microbeads containing 400 sorted endothelial cells per bead were seeded in fibrinogen solution combined with thrombin (0.625 U/ml). The microbeads were cultured with conditioned medium. The number and the length of the sprouts were recorded after 4 d of induction. The quantification of the sprouts was performed by using Image-Pro Plus 6.0.

**Western blots.**

After the cells were collected, the proteins were extracted using a lysis buffer (Beyotime); the cell lysates were resolved using sodium dodecyl sulfate–polyacrylamide gel electrophoresis (Bio-Rad Laboratories, Inc., Hercules, CA) and were electrophoretically transferred to nitrocellulose membranes (Bio-Rad Laboratories, Inc.). After blocking with 5% non-fat milk for 1 h at room temperature, the membranes were incubated with primary antibodies, including: Ezh2 (Cell Signaling, 1:2,000), Ezh1 (Abcam, 1:2,000), H3K27me3 (Cell Signaling, 1:2,000), p16INK4a (Abcam, 1:2,000), p21 (Abcam, 1:2,000), Ki67, (Novus Biologicals, 1:2,000), AMPK (Cell Signaling, 1:5,000), p-AMPK (Cell Signaling, 1:5,000), mTOR (Cell Signaling, 1:5,000), p-mTOR (Cell Signaling, 1:5,000), Hif1a (Cell Signaling, 1:2,000), VEGF (Cell Signaling, 1:5,000), and GAPDH (Abcam, 1:5,000). Proteins were visualized using SuperSignal West Dura Chemiluminescent Substrate (Pierce Biotechnology, Inc., Rockford, IL).

**ELISA analysis.**

We performed TGF-β1，PDGF-BB, IGF-1, FGF1 and VEGF ELISA analysis of bone marrow supernatant using a Mouse/Rat TGF-beta 1 Quantikine ELISA Kit (R&D Systems), Mouse/Rat PDGF-BB Quantikine ELISA kit (R&D Systems), Mouse/Rat IGF-I Quantikine ELISA Kit (R&D Systems), Mouse VEGF Quantikine ELISA Kit (R&D Systems) or Mouse FGF1 ELISA Kit (R&D Systems).
